# Synaptic density in carriers of C9orf72 mutations: a [^11^C]UCB‐J PET study

**DOI:** 10.1002/acn3.51407

**Published:** 2021-06-16

**Authors:** Maura Malpetti, Negin Holland, P. Simon Jones, Rong Ye, Thomas E. Cope, Tim D. Fryer, Young T. Hong, George Savulich, Timothy Rittman, Luca Passamonti, Elijah Mak, Franklin I. Aigbirhio, John T. O’Brien, James B. Rowe

**Affiliations:** ^1^ Department of Clinical Neurosciences University of Cambridge Cambridge UK; ^2^ Cambridge University Hospitals NHS Foundation Trust Cambridge UK; ^3^ Medical Research Council Cognition and Brain Sciences Unit University of Cambridge UK; ^4^ Wolfson Brain Imaging Centre University of Cambridge Cambridge UK; ^5^ Department of Psychiatry University of Cambridge Cambridge UK; ^6^ Istituto di Bioimmagini e Fisiologia Molecolare (IBFM) Consiglio Nazionale delle Ricerche (CNR) Milano Italy

## Abstract

Synaptic loss is an early and clinically relevant feature of many neurodegenerative diseases. Here we assess three adults at risk of frontotemporal dementia from C9orf72 mutation, using [^11^C]UCB‐J PET to quantify synaptic density in comparison with 19 healthy controls and one symptomatic patient with behavioural variant frontotemporal dementia. The three pre‐symptomatic C9orf72 carriers showed reduced synaptic density in the thalamus compared to controls, and there was an additional extensive synaptic loss in frontotemporal regions of the symptomatic patient. [^11^C]UCB‐J PET may facilitate early, pre‐symptomatic assessment, monitoring of disease progression and evaluation of new preventive treatment strategies for frontotemporal dementia.

## Introduction

Synaptic dysfunction and loss are early events in the pathogenesis of many neurodegenerative diseases. Frontotemporal dementia is associated with changes in behaviour and language in which post mortem human studies^1^, ^2^ and animal models^3^ suggest early and severe synaptic loss. In vivo methods for assessing synapse loss have recently emerged, including fluidic and radioligand biomarkers.^4^, ^5^

A fifth of frontotemporal dementia cases have autosomal dominant inheritance,^6^ most commonly due to hexanucleotide expansions in the *chromosome 9 open reading frame 72* (C9orf72), mutations of *granulin* (GRN), or *microtubule‐associated protein tau* (MAPT) genes. Familial frontotemporal dementias provide the opportunity to study early pathogenic processes, and evaluate biomarkers in the pre‐symptomatic phase of the disease. For example, pentraxin‐2, which is implicated in synaptic plasticity, is decreased in symptomatic C9orf72 mutation carriers, but not pre‐symptomatic carriers.^7^ However, such fluidic markers for synaptic loss do not elucidate in␣vivo spatial distributions of pathology, and the restricted spatial extent of pre‐symptomatic disease might reduce their sensitivity.

Previous volumetric analysis of C9orf72 mutation carriers indicated that the earliest changes are detected in the thalamus.^8^, ^9^, ^10^, ^11^ Here, we test the hypothesis that the earliest loss of synaptic density occurs in the thalamus. To do this, we used positron emission tomography (PET) with the radioligand [^11^C]UCB‐J, which binds to synaptic vesicle protein 2A, in three asymptomatic carriers of C9orf72 mutations, a patient with symptomatic frontotemporal dementia, and 19 healthy controls. Synaptic density was estimated through the determination of the binding potential of [^11^C]UCB‐J. This ligand offers a direct measure of synaptic density,^5^ reflects disease‐specific regional synaptic loss, and is related to other biomarkers and clinical severity.^12^, ^13^, ^14^, ^15^, ^16^

## Methods

### Participants

Three pre‐symptomatic carriers of C9orf72 mutations and one patient with a clinical diagnosis of probable behavioural variant of frontotemporal dementia (bvFTD)^17^ were recruited from the Cambridge Centre for Frontotemporal Dementia at the University of Cambridge. Nineteen healthy volunteers (8 females and 11 males; age range: 68.0 ± 7.0) were recruited from the UK National Institute for Health Research Join Dementia Research (JDR) register.

Demographic and clinical features are given in Table 1; individual age (mean age C9orf72 carriers = 50.3 ± 4.2) and sex are not reported to protect anonymity. The C9orf72 carriers were asymptomatic, with positive family histories, and were clinically unimpaired. Carrier 1 and Carrier 3 had a family history of genetic motor neuron disease, and at the time of PET they were approximately 20 years from the family average age of onset. Carrier 2 had a family history of genetic bvFTD and was ˜5 years from the family average age of onset. None of the carriers presented relevant clinical symptoms, scoring within the normal range on both the frontotemporal dementia rating scale and the amyotrophic lateral sclerosis rating scale (Table 1). However, all carriers underperformed in one or more frontal assessment tests, as compared to controls (Table 1). In particular, Carrier 3 underperformed on the revised version of Addenbrooke’s cognitive examination (ACE‐R, a cognitive screening test across five domains), and on working memory‐related tests. The patient with bvFTD presented with symptoms at the age of 58 manifesting as a progressive change in personality, apathy and executive dysfunction. There were initial cognitive deficits in executive tasks but with preservation of memory and visuospatial skills, praxis, semantics and normal pyramidal and extra‐pyramidal motor examinations; they were diagnosed at the age of 62. At the time of the PET scan (aged 63), they presented with a lack of insight and empathy, severe apathy, agrammatism and semantic language deficits, performing poorly in all cognitive domains, especially in executive tasks. No mutations were identified in C9orf72, GRN, MAPT or TBK1.

**Table 1:** Demographic and clinical characteristics.

	Controls (*N* = 19)	Carrier 1	Carrier 2	Carrier 3	bvFTD patient
Gene mutation	—	C9orf72	C9orf72	C9orf72	—
Symptom duration (years)	—	—	—	—	4.5
Education (years)	13.4 ± 2.7	13 *(*−*0.1)*	11 *(*−*0.9)*	>20 *(>2.5)*°	10 *(*−*1.3)*
MMSE [max 30]	29.4 ± 1.2	28 *(*−*1.2)*	30 *(0.5)*	29 *(*−*0.3)*	14 *(*−12.7)^*^
ACE‐R [max 100]	96.1 ± 2.8	95 *(*−*0.4)*	100 *(1.4)*	90 *(*−2.2)^*^	46 *(*−18.0)^*^
Attent/Orient [max 18]	17.9 ± 0.3	17 *(*−2.8)^*^	18 *(0.3)*	17 *(*−2.8)^*^	6 *(*−37.7)^*^
Memory [max 26]	24.5 ± 1.8	26 *(0.9)*	26 *(0.9)*	21 *(*−2.0)^*^	12 *(*−7.0)^*^
Fluency [max 14]	12.5 ± 1.5	11 *(*−*1.0)*	14 *(1.0)*	12 *(*−*0.3)*	1 *(*−7.8)^*^
Language [max 26]	25.6 ± 0.8	25 *(*−*0.7)*	26 *(0.5)*	25 *(*−*0.7)*	21 *(*−5.5)^*^
Visuospatial [max 16]	15.7 ± 0.6	16 *(0.5)*	16 *(0.5)*	15 *(*−*1.2)*	6 *(*−16.6)^*^
INECO [max 30]	25.6 ± 2.0	26 *(0.2)*	22 *(*−1.8)^*^	25 *(*−*0.3)*	5 *(*−10.1) ^*^
INECO wm [max 10]	7.3 ± 1.3	8 *(0.5)*	7 *(*−*0.3)*	5 *(*−1.8)^*^	3 *(*−3.4) ^*^
FTD‐RS (%)	—	100%	100%	100%	11.5%
ALS‐RS (%)	—	100%	100%	100%	—

For controls, group mean and standard deviation are reported, while for each carrier and the bvFTD patients *z* scores versus controls are reported in brackets (*z < −1.645, one‐tailed test *p* < 0.05; *°z* < −1.96 or *z* > 1.96, two‐tailed test *p* < 0.05).

Abbreviations: ACE‐R, Addenbrooke's cognitive examination revised; ALS‐RS, amyotrophic lateral sclerosis rating scale; Attent/orient, attention/orientation score; bvFTD, behavioural variant of frontotemporal dementia; FTD‐RS, frontotemporal dementia rating scale; max, maximum; MMSE, mini‐mental state examination; wm, working memory.

The research protocol was approved by the Cambridge Research Ethics Committee (REC: 18/EE/0059) and the Administration of Radioactive Substances Advisory Committee. All participants provided written informed consent in accordance with the Declaration of Helsinki.

### Data acquisition and PET kinetic analysis

Participants underwent clinical and neuropsychological assessment, and brain imaging with 3T MRI and PET scanning with [^11^C]UCB‐J ((*R*)‐1‐((3‐(methyl‐11C)pyridin‐4‐yl)methyl)‐4‐(3,4,5‐trifluorophenyl)pyr‐rolidin‐2‐one). Dynamic PET data acquisition was performed on a GE SIGNA PET/MR (GE Healthcare, Waukesha, USA) for 90 min following [^11^C]UCB‐J injection (median injected activity 334 MBq), with attenuation correction including the use of a multi‐subject atlas method^17^ and improvements to the brain MRI coil component.^18^ Each emission image series was aligned and rigidly registered to T1‐weighted MRI acquired in the same session (TR = 3.6 msec, TE = 9.2 msec, 192 sagittal slices, in‐plane voxel dimensions 0.55 × 0.55 mm (subsequently interpolated to 1.0 × 1.0 mm); slice thickness 1.0 mm).

To facilitate voxel‐wise statistical testing, for each subject a [^11^C]UCB‐J non‐displaceable binding potential (BP_ND_) map was determined from dynamic images corrected for partial volume error at the voxel level using the iterative Yang method.^19^ BP_ND_ was calculated using a basis function implementation of the simplified reference tissue model, with centrum semiovale as the reference tissue.^20^ Each BP_ND_ map was warped to the ICBM 152 2009a asymmetric MR template using parameters from the spatial normalisation of the co‐registered T1 MR image with Advanced Normalization Tools (ANTs; http://www.picsl.upenn.edu/ANTS/). BP_ND_ was quantified in sub‐divisions of the thalamus by warping the scale IV ROIs from the Melbourne subcortex atlas^21^ to the ICBM 152 2009a asymmetric MR template and extracting values from the partial volume corrected BP_ND_ maps.

For whole‐brain regional analysis, a version of the n30r83 Hammers atlas (http://brain‐development.org) modified to include segmentation of posterior fossa regions was spatially normalised to the T1‐weighted MRI of each participant. Regions were multiplied by a binary grey matter mask (>50% on the SPM12 grey matter probability map smoothed to PET spatial resolution) and geometric transfer matrix partial volume correction^19^ was applied to each image of the dynamic series. Regional BP_ND_ was determined using the same reference tissue approach as for the BP_ND_ maps.

### Statistical analysis

To test our primary hypothesis, we assessed thalamic synaptic density. We compared the unsmoothed partial‐volume corrected BP_ND_ map of each carrier versus controls, using the *randomise* function in FSL (version 5.0.10) within a thalamic regional mask to generate a voxel‐wise non‐parametric *t*‐test (Conditional Monte Carlo permutation test, 5000 permutations). Cluster significance was defined at *p* < 0.05 family‐wise error rate (FWE) and clusters identified using the threshold‐free cluster estimation method. We also calculated *z*‐scores for BP_ND_ in the thalamic sub‐regions in comparison to control data.

Next, we performed exploratory, illustrative analyses across the whole brain using smoothed BP_ND_ maps (isotropic 6 mm full width at half maximum Gaussian). First, for the patient and each mutation carrier, we calculated voxel‐wise *z*‐score maps of BP_ND_, to reveal voxels with reduced BP_ND_ in each carrier/patient. Next, we calculated BP_ND_ *z*‐scores for each region of the modified Hammers atlas. In both cases, we considered a statistical threshold of *z* = −1.645 (*p* < 0.05 uncorrected) which corresponds to the 95th percentile of the normal distribution for a one‐tailed test, based on the hypothesis of reduced synaptic density in patients and carriers compared to controls.

## Results

**FIGURE 1:**
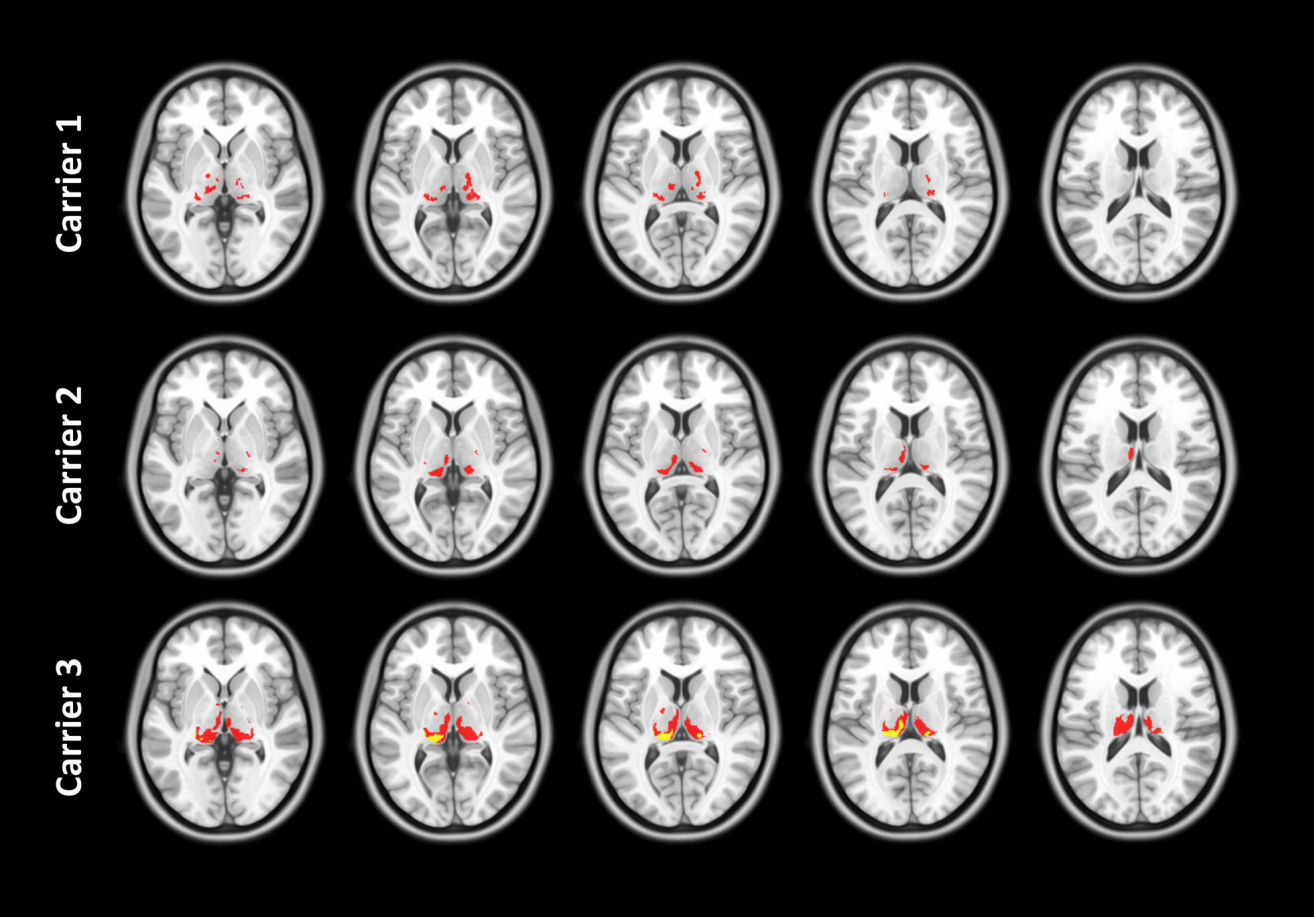
Thalamic synaptic loss in C9orf72 carriers. Results for voxel‐wise [^11^C]UCB‐J BP_ND_ comparisons for each carrier versus controls using the FSL randomise function. Results are reported with significance *p* < 0.05 with family‐wise error (FWE) correction at cluster level (yellow) and *p* < 0.05 uncorrected for multiple comparisons at voxel level (red).

Reduction of thalamic [^11^C]UCB‐J BP_ND_ was seen in each C9orf72 carrier, in posterior, pulvinar and lateral regions (Fig. 1 – significance *p* < 0.05 with FWE correction at cluster level and *p* < 0.05 uncorrected for multiple comparisons at voxel level). Thalamic sub‐region BP_ND_ and *z*‐scores are presented in Table S1.

**FIGURE 2:**
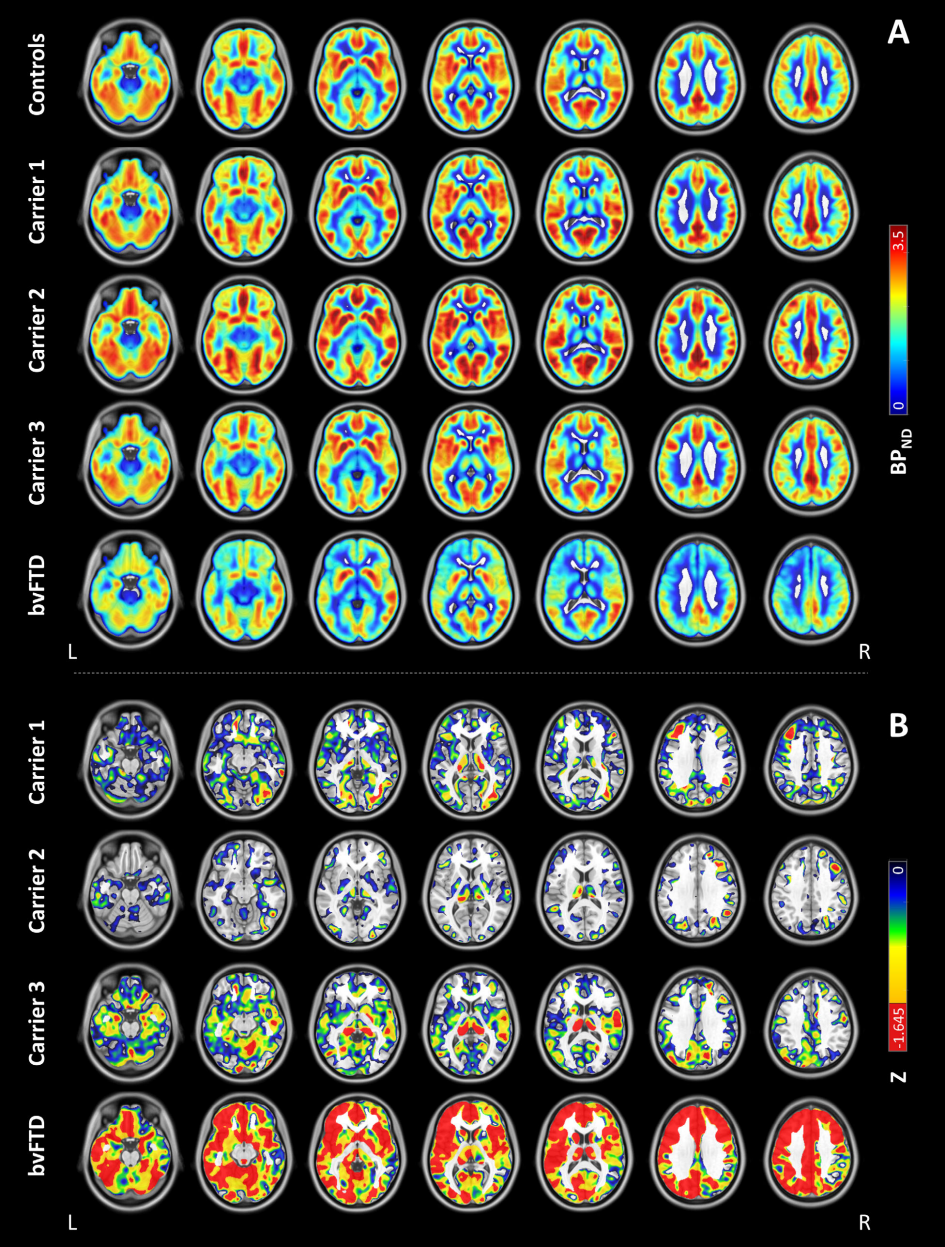
Whole‐brain voxel‐wise binding potential (BP_ND_) maps and *z*‐score maps. (A) Axial slices of [^11^C]UCB‐J BP_ND_ maps for control mean (first row), the three carriers (rows 2–4) and the bvFTD patient (fifth row). High and low BP_ND_ values are shown by red and blue areas respectively. (B) Axial slices of voxel‐wise negative *z*‐score maps (rows 6–9), with voxel *z*‐scores <−1.645 highlighted in red (*p* < 0.05). All slices are reported in the neurological display convention (left on the left) and maps are overlaid on the ICBM 152 2009a asymmetric T1 MR template.

Single subject and control mean [^11^C]UCB‐J BP_ND_ maps are displayed in Figure 2 (panel A), with individual *z*‐score maps for the three mutation carriers and patient (panel B). The participant with bvFTD showed widespread severe reduction of synaptic density, especially in the left frontal and temporal cortex, subcortical regions and, to a lesser extent, parietal cortex. For all C9orf72 mutation carriers, the most prominent reduction in [^11^C]UCB‐J binding was evident in the thalamus, along with a scattered and lesser reduction in cortical regions.

**FIGURE 3:**
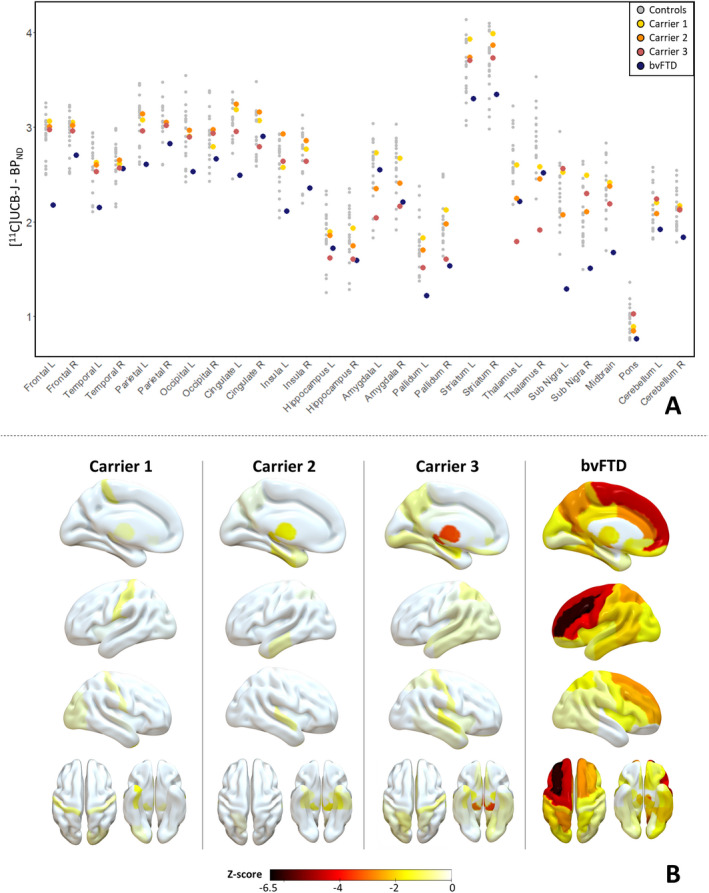
Regional binding potential (BP_ND_) values and *z*‐scores. (A) Individual regional [^11^C]UCB‐J BP_ND_ values for controls (grey), carriers (yellow, orange and coral) and the bvFTD patient (blue). Aggregated values are displayed for frontal, temporal, parietal, and occipital lobes, cingulate and cerebellum. (B) Individual negative regional *z*‐score scores displayed on a glass brain for each of three carriers (columns 1–3) and the bvFTD patient (righthand column).

Regional [^11^C]UCB‐J BP_ND_ values for the Hammers atlas are reported in Figure 3 (panel A), together with rendering of individual regional *z*‐scores (panel B). The bvFTD patient showed reduced [^11^C]UCB‐J binding in the left frontal, temporal and parietal regions, cingulum, insula and thalamus, and bilaterally in middle and superior frontal gyri, the superior parietal gyrus, pallidum and substantia nigra. The C9orf72 mutation carriers had reduced [^11^C]UCB‐J binding in thalamus, reaching statistical threshold in Carrier 2 (left *z* = −1.77; right *z* = −1.26) and Carrier 3 (left *z* = −3.46; right *z* = −2.99). A significant reduction of [^11^C]UCB‐J binding was also found in the cerebellar dentate nucleus of Carrier 1 (right *z* = −1.77) and Carrier 2 (right *z* = −2.25), and in the pre‐subgenual frontal cortex of Carrier 3 (right *z* = −1.71). Regional BP_ND_ values and *z*‐scores are tabulated in Table S2.

## Discussion

Each of three pre‐symptomatic carriers of pathogenic mutations in C9orf72 showed in␣vivo evidence of synaptic loss in the thalamus. The restricted thalamic reduction in pre‐symptomatic carriers contrasts with the extensive cortical and subcortical synaptic loss in a patient with symptomatic frontotemporal dementia.

In the C9orf72 carriers, synaptic density was most reduced in the ventral‐posterior thalamic sub‐region and the pulvinar nuclei (dorsal‐posterior thalamic sub‐region). Despite age differences between carriers and controls, previous evidence suggests that [^11^C]UCB‐J binding reflecting synaptic density does not decline with age.^22^ In familial FTD, thalamic atrophy has been commonly reported in C9orf72 mutation carriers,^9^, ^10^, ^23^, ^24^, ^25^, ^26^ early in the pre‐symptomatic stage before the age of 40^11^ and decades prior to symptom onset.^8^ Thalamic neurodegeneration may not be specific to C9orf72 mutations. Thalamic atrophy can occur across the whole spectrum of frontotemporal dementia,^27^, ^28^, ^29^, ^30^ especially in those caused by TDP‐43 pathology.^31^ However, among familial frontotemporal dementia, those with C9orf72 mutation show the earliest and the most severe thalamic volume loss. Our finding, with partial volume correction applied to counter the known effect of thalamic atrophy, is consistent with a selective involvement of the pulvinar nucleus in C9orf72 expansion carriers.^10^ The [^11^C]UCB‐J PET findings also accord with pathological evidence of RNA foci, dipeptide repeat protein inclusions and TDP‐43 pathology in the thalamus.^32^ The functional significance is suggested by the correlation between pulvinar atrophy and salience network connectivity in patients with C9orf72 mutations.^24^

Carriers presented with a heterogenous family and clinical profile. Two carriers had a family history of motor neuron disease, and one of bvFTD. Carrier 3 underperformed in frontal tests, and presented the most severe pattern of synaptic loss in thalamic regions, despite being the carrier with the highest number of years of education and estimated years from onset; albeit it is well established that estimated years of symptom onset, deduced from affected family members, is a weak predictor of age of onset in C9orf72 carriers.^33^ Carrier 3 also obtained low memory ACE‐R sub‐score, which is not a typical first domain of impairment in the FTD/ALS spectrum, although is common later in disease. Despite this heterogeneity, synaptic loss in the thalamus was seen across all subjects. Comparing our findings with those from [^11^C]UCB‐J PET in symptomatic C9orf72 carriers will help to further clarify the longitudinal impact of early thalamic impairment.

In summary, this study indicates that thalamic synaptic loss occurs early in C9orf72, before symptom onset, with a thalamic focus that has the potential to disrupt connectivity in multiple neural circuits and spread to widespread cortical regions. We suggest that [^11^C]UCB‐J is a useful biomarker for in␣vivo quantification of synaptic loss in frontotemporal dementia. Further investigations with other genetic aetiologies would be required to examine the specificity of these effects to C9orf72 mutations.

## Conflict of Interest

All authors have no conflicts of interest. Unrelated to this work, JBR serves as an associate editor to Brain and is a non‐remunerated trustee of the Guarantors of Brain, Darwin College and the PSP Association (UK). He provides consultancy to Asceneuron, Biogen, UCB and has research grants from AZ‐Medimmune, Janssen and Lilly as industry partners in the Dementias Platform UK. TR has received honoraria from Biogen, Oxford Biomedica and the National Institute for Health and Clinical Excellence (NICE). JTO has received honoraria for work as DSMB chair or member for TauRx, Axon, Eisai and Novo Nordisk and, has acted as a consultant for Biogen, Roche, and has received research support from Alliance Medical and Merck.

## Supporting information


**Table␣S1**. Thalamic sub‐division regional [^11^C]UCB‐J binding potential values (BP_ND_) and *z*‐scores (Z) for each carrier and the bvFTD patient versus controls. Regions with *z*‐scores <−1.645, corresponding to the 95th percentile of a normal distribution for a one‐tailed test, are shown in red font.
**Table␣S2**. Whole‐brain regional [^11^C]UCB‐J binding potential values (BP) and *z*‐scores (Z) for each carrier and the bvFTD patient versus controls. Regions with *z*‐scores <−1.645, corresponding to the 95th percentile of a normal distribution for a one‐tailed test, are shown in red font.
