# Supplementary material for: Synaptic density in carriers of C9orf72 mutations: a [11C]UCB‐J PET study
Source: Ann Clin Transl Neurol. 2021 Jun 16;8(7):1515–23. doi: 10.1002/acn3.51407 (PMC8283163; doi:10.1002/acn3.51407)
Supplement: Supplementary file 1 — Table␣S1. Thalamic sub‐division regional [11C]UCB‐J binding potential values (BPND) and z‐scores (Z) for each carrier and the bvFTD patient versus controls. Regions with z‐scores <−1.645, corresponding to the 95th percentile of a normal distribution for a one‐tailed test, are shown in red font. Table␣S2. Whole‐brain regional [11C]UCB‐J binding potential values (BP) and z‐scores (Z) for each carrier and the bvFTD patient versus controls. Regions with z‐scores <−1.645, corresponding to the 95th percentile of a normal distribution for a one‐tailed test, are shown in red font. [file ACN3-8-1515-s001.docx]

**Supplementary material**

**Supplementary Table 1. Thalamic sub-division regional [^11^C]UCB-J binding potential values (BP_ND_) and z-scores (Z) for each carrier and the bvFTD patient versus controls.** Regions with z-scores < -1.645, corresponding to the 95th percentile of a normal distribution for a one-tailed test, are shown in red font.

| Thalamic sub-region | Controls | | Carrier 1 | | Carrier 2 | | Carrier 3 | | | bvFTD | | |
| --- | --- | --- | --- | --- | --- | --- | --- | --- | --- | --- | --- | --- |
|  | **Mean** | **SD** | **BP_ND_** | **Z** | **BP_ND_** | **Z** | **BP_ND_** | **Z** | **BP_ND_** | | **Z** |  |
| Inferior ventroanterior - anterior division R | 1.88 | 0.26 | 1.65 | -0.89 | 1.76 | -0.49 | 1.55 | -1.29 | 1.56 | | -1.27 |  |
| Inferior ventroanterior - posterior division R | 1.29 | 0.26 | 1.22 | -0.27 | 1.39 | 0.37 | 1.15 | -0.55 | 0.88 | | -1.59 |  |
| Superior ventroanterior R | 1.44 | 0.25 | 1.30 | -0.53 | 1.34 | -0.40 | 1.24 | -0.77 | 0.95 | | -1.95^*^ |  |
| Medial ventroposterior R | 2.18 | 0.24 | 2.04 | -0.58 | 1.96 | -0.94 | 1.63 | -2.31^*^ | 2.03 | | -0.61 |  |
| Lateral ventroposterior R | 1.91 | 0.23 | 1.50 | -1.77^*^ | 1.72 | -0.83 | 1.63 | -1.20 | 1.73 | | -0.79 |  |
| Medial dorsoanterior R | 1.94 | 0.25 | 1.97 | 0.14 | 1.65 | -1.14 | 1.22 | -2.81^*^ | 1.52 | | -1.64 |  |
| Lateral dorsoanterior R | 1.75 | 0.27 | 1.38 | -1.36 | 1.63 | -0.44 | 1.37 | -1.42 | 1.36 | | -1.45 |  |
| Dorsoposterior R | 1.95 | 0.21 | 1.67 | -1.32 | 1.78 | -0.82 | 1.31 | -3.02^*^ | 1.70 | | -1.21 |  |
| Inferior ventroanterior - anterior division L | 1.82 | 0.26 | 1.64 | -0.66 | 1.61 | -0.80 | 1.52 | -1.13 | 1.38 | | -1.69^*^ |  |
| Inferior ventroanterior - posterior division L | 1.19 | 0.23 | 1.19 | -0.01 | 1.11 | -0.36 | 1.01 | -0.80 | 0.86 | | -1.46 |  |
| Superior ventroanterior L | 1.44 | 0.23 | 1.35 | -0.38 | 1.21 | -0.98 | 1.07 | -1.60 | 0.97 | | -2.03^*^ |  |
| Medial ventroposterior L | 2.23 | 0.22 | 2.07 | -0.72 | 1.95 | -1.24 | 1.67 | -2.53^*^ | 2.00 | | -1.01 |  |
| Lateral ventroposterior L | 1.83 | 0.23 | 1.44 | -1.68^*^ | 1.70 | -0.53 | 1.42 | -1.76^*^ | 1.49 | | -1.45 |  |
| Medial dorsoanterior L | 2.10 | 0.26 | 2.08 | -0.07 | 1.66 | -1.73^*^ | 1.24 | -3.35^*^ | 1.50 | | -2.32^*^ |  |
| Lateral dorsoanterior L | 1.78 | 0.29 | 1.44 | -1.13 | 1.49 | -0.95 | 0.94 | -2.83^*^ | 1.28 | | -1.67^*^ |  |
| Dorsoposterior L | 1.96 | 0.20 | 1.77 | -0.90 | 1.69 | -1.31 | 1.15 | -3.96^*^ | 1.42 | | -2.62^*^ |  |

Abbreviations: L: left; R: right.

**Supplementary Table 2. Whole-brain regional [^11^C]UCB-J binding potential values (BP) and z-scores (Z) for each carrier and the bvFTD patient versus controls.** Regions with z-scores < -1.645, corresponding to the 95th percentile of a normal distribution for a one-tailed test, are shown in red font.

| Region | Controls | | Carrier 1 | | Carrier 2 | | Carrier 3 | | bvFTD | | |
| --- | --- | --- | --- | --- | --- | --- | --- | --- | --- | --- | --- |
|  | **Mean** | **SD** | **BP_ND_** | **Z** | **BP_ND_** | **Z** | **BP_ND_** | **Z** | **BP_ND_** | **Z** |  |
| Hippocampus_R | 1.89 | 0.29 | 1.93 | 0.16 | 1.74 | -0.49 | 1.61 | -0.96 | 1.59 | -1.00 |  |
| Hippocampus_L | 1.89 | 0.28 | 1.89 | 0.03 | 1.86 | -0.10 | 1.62 | -0.94 | 1.72 | -0.59 |  |
| Amygdala_R | 2.57 | 0.30 | 2.67 | 0.33 | 2.41 | -0.55 | 2.16 | -1.36 | 2.21 | -1.21 |  |
| Amygdala_L | 2.52 | 0.33 | 2.73 | 0.62 | 2.35 | -0.52 | 2.04 | -1.46 | 2.55 | 0.07 |  |
| Anterior_temporal_lobe_medial_part_R | 2.21 | 0.22 | 1.88 | -1.50 | 2.11 | -0.46 | 2.00 | -0.95 | 2.18 | -0.17 |  |
| Anterior_temporal_lobe_medial_part_L | 2.20 | 0.22 | 2.20 | 0.01 | 2.08 | -0.52 | 2.20 | -0.01 | 2.08 | -0.55 |  |
| Anterior_temporal_lobe_lateral_part_R | 2.51 | 0.25 | 2.59 | 0.33 | 2.51 | -0.02 | 2.50 | -0.04 | 2.65 | 0.54 |  |
| Anterior_temporal_lobe_lateral_part_L | 2.54 | 0.31 | 2.63 | 0.29 | 2.52 | -0.04 | 2.56 | 0.08 | 2.18 | -1.15 |  |
| Parahippocampal_and_ambient_gyri_R | 1.74 | 0.24 | 1.73 | -0.06 | 1.58 | -0.65 | 1.73 | -0.06 | 1.62 | -0.51 |  |
| Parahippocampal_and_ambient_gyri_L | 1.52 | 0.26 | 1.75 | 0.87 | 1.30 | -0.86 | 1.56 | 0.15 | 1.41 | -0.42 |  |
| Superior_temporal_gyrus_posterior_part_R | 2.85 | 0.19 | 2.82 | -0.16 | 2.72 | -0.70 | 2.64 | -1.11 | 2.90 | 0.25 |  |
| Superior_temporal_gyrus_posterior_part_L | 2.78 | 0.31 | 2.79 | 0.01 | 2.87 | 0.28 | 2.67 | -0.37 | 2.42 | -1.19 |  |
| Middle_and_inferior_temporal_gyrus_R | 2.69 | 0.25 | 2.75 | 0.28 | 2.73 | 0.17 | 2.62 | -0.26 | 2.72 | 0.14 |  |
| Middle_and_inferior_temporal_gyrus_L | 2.70 | 0.31 | 2.73 | 0.10 | 2.55 | -0.51 | 2.57 | -0.42 | 2.12 | -1.91^*^ |  |
| Fusiform_gyrus_R | 2.46 | 0.29 | 2.12 | -1.16 | 2.25 | -0.70 | 2.18 | -0.97 | 2.30 | -0.53 |  |
| Fusiform_gyrus_L | 2.43 | 0.25 | 2.69 | 1.04 | 2.10 | -1.34 | 2.27 | -0.65 | 2.03 | -1.63 |  |
| Insula_L | 2.60 | 0.27 | 2.58 | -0.10 | 2.93 | 1.22 | 2.64 | 0.13 | 2.12 | -1.82^*^ |  |
| Insula_R | 2.72 | 0.25 | 2.77 | 0.20 | 2.86 | 0.55 | 2.64 | -0.31 | 2.36 | -1.42 |  |
| Lateral_remainder_of_occipital_lobe_L | 2.76 | 0.29 | 2.75 | -0.04 | 2.80 | 0.13 | 2.68 | -0.30 | 2.31 | -1.56 |  |
| Lateral_remainder_of_occipital_lobe_R | 2.71 | 0.25 | 2.62 | -0.38 | 2.78 | 0.26 | 2.74 | 0.08 | 2.58 | -0.54 |  |
| Cingulate_gyrus_anterior_part_L | 2.89 | 0.24 | 3.04 | 0.64 | 3.19 | 1.23 | 2.87 | -0.07 | 2.22 | -2.79^*^ |  |
| Cingulate_gyrus_anterior_part_R | 2.83 | 0.27 | 2.94 | 0.40 | 2.94 | 0.39 | 2.66 | -0.64 | 2.61 | -0.81 |  |
| Gyrus_cinguli_posterior_part_L | 3.03 | 0.24 | 3.18 | 0.64 | 3.25 | 0.94 | 2.98 | -0.20 | 2.57 | -1.94^*^ |  |
| Gyrus_cinguli_posterior_part_R | 3.03 | 0.25 | 3.11 | 0.35 | 3.37 | 1.37 | 2.92 | -0.41 | 2.93 | -0.39 |  |
| Middle_frontal_gyrus_L | 2.92 | 0.26 | 2.96 | 0.14 | 3.03 | 0.42 | 2.99 | 0.27 | 1.36 | -6.08^*^ |  |
| Middle_frontal_gyrus_R | 2.97 | 0.25 | 2.99 | 0.07 | 3.12 | 0.62 | 3.01 | 0.17 | 2.35 | -2.45^*^ |  |
| Posterior_temporal_lobe_L | 2.72 | 0.28 | 2.74 | 0.05 | 2.80 | 0.28 | 2.61 | -0.40 | 2.14 | -2.12^*^ |  |
| Posterior_temporal_lobe_R | 2.71 | 0.25 | 2.77 | 0.22 | 2.81 | 0.39 | 2.68 | -0.15 | 2.59 | -0.48 |  |
| Inferiolateral_remainder_of_parietal_lobe_L | 2.87 | 0.27 | 2.95 | 0.32 | 3.14 | 1.01 | 2.79 | -0.28 | 2.32 | -2.00^*^ |  |
| Inferiolateral_remainder_of_parietal_lobe_R | 2.83 | 0.24 | 2.92 | 0.38 | 2.93 | 0.44 | 2.92 | 0.37 | 2.65 | -0.74 |  |
| Caudate_nucleus_L | 3.04 | 0.35 | 3.34 | 0.85 | 3.28 | 0.69 | 3.16 | 0.33 | 2.92 | -0.36 |  |
| Caudate_nucleus_R | 3.02 | 0.36 | 3.38 | 1.00 | 3.27 | 0.70 | 3.17 | 0.43 | 2.99 | -0.08 |  |
| Nucleus_accumbens_L | 3.81 | 0.31 | 4.33 | 1.69 | 3.80 | -0.01 | 3.89 | 0.27 | 3.33 | -1.56 |  |
| Nucleus_accumbens_R | 4.01 | 0.39 | 4.57 | 1.44 | 4.24 | 0.58 | 4.07 | 0.16 | 3.46 | -1.41 |  |
| Putamen_L | 3.96 | 0.40 | 4.12 | 0.41 | 4.12 | 0.40 | 4.06 | 0.26 | 3.66 | -0.74 |  |
| Putamen_R | 3.88 | 0.37 | 4.01 | 0.37 | 4.09 | 0.58 | 3.95 | 0.21 | 3.59 | -0.77 |  |
| Thalamus_L | 2.72 | 0.27 | 2.60 | -0.46 | 2.25 | -1.77^*^ | 1.79 | -3.46^*^ | 2.21 | -1.88^*^ |  |
| Thalamus_R | 2.84 | 0.31 | 2.58 | -0.84 | 2.45 | -1.26 | 1.91 | -2.99^*^ | 2.52 | -1.05 |  |
| Pallidum_L | 1.76 | 0.28 | 1.83 | 0.26 | 1.70 | -0.19 | 1.52 | -0.85 | 1.22 | -1.90^*^ |  |
| Pallidum_R | 1.99 | 0.25 | 2.13 | 0.53 | 1.98 | -0.06 | 1.60 | -1.54 | 1.53 | -1.82^*^ |  |
| Precentral_gyrus_L | 2.69 | 0.19 | 2.73 | 0.24 | 2.94 | 1.34 | 2.81 | 0.65 | 1.83 | -4.43^*^ |  |
| Precentral_gyrus_R | 2.67 | 0.20 | 2.68 | 0.08 | 2.73 | 0.29 | 2.70 | 0.15 | 2.38 | -1.43 |  |
| Straight_gyrus_L | 2.79 | 0.31 | 2.86 | 0.23 | 3.00 | 0.66 | 2.57 | -0.70 | 2.22 | -1.82^*^ |  |
| Straight_gyrus_R | 2.73 | 0.27 | 2.77 | 0.13 | 2.97 | 0.84 | 2.60 | -0.49 | 2.44 | -1.09 |  |
| Anterior_orbital_gyrus_L | 2.74 | 0.25 | 3.01 | 1.08 | 2.77 | 0.12 | 2.83 | 0.36 | 2.48 | -1.04 |  |
| Anterior_orbital_gyrus_R | 2.80 | 0.25 | 2.88 | 0.31 | 3.00 | 0.83 | 3.03 | 0.92 | 3.17 | 1.51 |  |
| Inferior_frontal_gyrus_L | 2.85 | 0.26 | 2.96 | 0.42 | 3.00 | 0.57 | 3.04 | 0.71 | 1.78 | -4.15^*^ |  |
| Inferior_frontal_gyrus_R | 2.86 | 0.22 | 2.94 | 0.36 | 2.93 | 0.29 | 3.04 | 0.81 | 2.59 | -1.23 |  |
| Superior_frontal_gyrus_L | 2.88 | 0.26 | 3.03 | 0.57 | 2.98 | 0.40 | 2.94 | 0.23 | 1.72 | -4.54^*^ |  |
| Superior_frontal_gyrus_R | 2.91 | 0.27 | 3.17 | 0.94 | 3.03 | 0.43 | 2.90 | -0.04 | 2.17 | -2.74^*^ |  |
| Postcentral_gyrus_L | 2.73 | 0.24 | 2.54 | -0.81 | 2.85 | 0.48 | 2.70 | -0.15 | 2.21 | -2.17^*^ |  |
| Postcentral_gyrus_R | 2.70 | 0.24 | 2.57 | -0.56 | 2.73 | 0.11 | 2.51 | -0.79 | 2.53 | -0.71 |  |
| Superior_parietal_gyrus_L | 3.15 | 0.28 | 3.20 | 0.16 | 3.09 | -0.21 | 2.97 | -0.66 | 2.41 | -2.65^*^ |  |
| Superior_parietal_gyrus_R | 3.11 | 0.25 | 3.14 | 0.13 | 3.22 | 0.43 | 3.03 | -0.31 | 2.68 | -1.67^*^ |  |
| Lingual_gyrus_L | 2.87 | 0.35 | 2.98 | 0.32 | 3.10 | 0.64 | 2.64 | -0.66 | 2.70 | -0.49 |  |
| Lingual_gyrus_R | 2.98 | 0.30 | 3.00 | 0.10 | 3.17 | 0.66 | 2.94 | -0.13 | 2.75 | -0.75 |  |
| Cuneus_L | 3.13 | 0.39 | 3.14 | 0.04 | 3.11 | -0.05 | 3.15 | 0.05 | 2.54 | -1.52 |  |
| Cuneus_R | 3.17 | 0.37 | 3.00 | -0.48 | 3.28 | 0.29 | 3.18 | 0.01 | 2.75 | -1.16 |  |
| Medial_orbital_gyrus_L | 2.63 | 0.30 | 2.89 | 0.85 | 2.92 | 0.96 | 2.59 | -0.13 | 2.21 | -1.41 |  |
| Medial_orbital_gyrus_R | 2.66 | 0.31 | 2.80 | 0.45 | 2.92 | 0.83 | 2.53 | -0.40 | 2.68 | 0.07 |  |
| Lateral_orbital_gyrus_L | 2.33 | 0.29 | 2.61 | 1.00 | 2.74 | 1.44 | 2.67 | 1.19 | 1.87 | -1.58 |  |
| Lateral_orbital_gyrus_R | 2.43 | 0.27 | 2.53 | 0.37 | 2.66 | 0.85 | 2.57 | 0.52 | 2.62 | 0.71 |  |
| Posterior_orbital_gyrus_L | 2.53 | 0.25 | 2.72 | 0.74 | 2.91 | 1.51 | 2.71 | 0.73 | 2.21 | -1.24 |  |
| Posterior_orbital_gyrus_R | 2.54 | 0.24 | 2.69 | 0.64 | 2.78 | 0.99 | 2.75 | 0.87 | 2.49 | -0.23 |  |
| Substantia_nigra_L | 2.26 | 0.30 | 2.52 | 0.89 | 2.07 | -0.63 | 2.56 | 1.02 | 1.29 | -3.27^*^ |  |
| Substantia_nigra_R | 2.08 | 0.32 | 2.49 | 1.30 | 2.11 | 0.08 | 2.30 | 0.69 | 1.51 | -1.81^*^ |  |
| Subgenual_frontal_cortex_L | 2.74 | 0.28 | 2.69 | -0.17 | 3.00 | 0.91 | 2.85 | 0.38 | 2.42 | -1.16 |  |
| Subgenual_frontal_cortex_R | 2.62 | 0.40 | 2.46 | -0.38 | 2.71 | 0.22 | 2.10 | -1.30 | 2.27 | -0.86 |  |
| Subcallosal_area_L | 3.16 | 0.62 | 3.15 | -0.01 | 3.52 | 0.59 | 3.06 | -0.15 | 2.20 | -1.53 |  |
| Subcallosal_area_R | 2.96 | 0.64 | 2.70 | -0.41 | 3.48 | 0.81 | 2.57 | -0.61 | 2.74 | -0.34 |  |
| Presubgenual_frontal_cortex_L | 3.44 | 0.39 | 3.92 | 1.24 | 3.68 | 0.63 | 3.17 | -0.70 | 3.04 | -1.02 |  |
| Presubgenual_frontal_cortex_R | 3.31 | 0.43 | 3.51 | 0.47 | 3.30 | -0.03 | 2.58 | -1.71^*^ | 3.06 | -0.59 |  |
| Superior_temporal_gyrus_anterior_part_L | 2.27 | 0.27 | 2.40 | 0.49 | 2.42 | 0.56 | 2.13 | -0.53 | 2.03 | -0.87 |  |
| Superior_temporal_gyrus_anterior_part_R | 2.37 | 0.28 | 2.36 | -0.05 | 2.37 | -0.03 | 2.24 | -0.48 | 2.52 | 0.53 |  |
| Brainstem_midbrain | 2.31 | 0.30 | 2.41 | 0.36 | 2.37 | 0.22 | 2.19 | -0.40 | 1.67 | -2.11^*^ |  |
| Brainstem_pons | 0.96 | 0.16 | 0.89 | -0.42 | 0.85 | -0.69 | 1.03 | 0.41 | 0.76 | -1.25 |  |
| Brainstem_medulla | 0.67 | 0.15 | 0.72 | 0.30 | 0.75 | 0.50 | 0.73 | 0.41 | 0.53 | -1.01 |  |
| Cerebellum_grey-matter_R | 2.13 | 0.22 | 2.17 | 0.20 | 2.13 | 0.01 | 2.10 | -0.13 | 1.83 | -1.34 |  |
| Cerebellum_grey-matter_L | 1.23 | 0.72 | 2.19 | 1.34 | 2.07 | 1.18 | 2.21 | 1.37 | 1.91 | 0.95 |  |
| Cerebellum_dentate_L | 1.04 | 0.17 | 1.07 | 0.17 | 1.10 | 0.33 | 1.23 | 1.05 | 0.63 | -2.36^*^ |  |
| Cerebellum_dentate_R | 2.02 | 0.56 | 1.03 | -1.77^*^ | 0.77 | -2.25^*^ | 1.16 | -1.54 | 0.61 | -2.54^*^ |  |

Abbreviations: L: left; R: right.
